# Correction: Cesium carbonate mediated C–H functionalization of perhalogenated 12-vertex carborane anions

**DOI:** 10.1039/d2cc90180c

**Published:** 2022-05-24

**Authors:** Sergio O. Lovera, Alex L. Bagdasarian, Juchen Guo, Hosea M. Nelson, Vincent Lavallo

**Affiliations:** Departments of Chemistry, University of California Riverside Riverside CA 92521 USA vincent.lavallo@ucr.edu; Department of Chemistry and Chemical Engineering, California Institute of Technology Pasadena CA 91125 USA hosea@caltech.edu http://www.thenelsonlab.com; Department of Chemical and Environmental Engineering, University of California Riverside Riverside CA 92521 USA jguo@engr.ucr.edu

## Abstract

Correction for ‘Cesium carbonate mediated C–H functionalization of perhalogenated 12-vertex carborane anions’ by Sergio O. Lovera *et al.*, *Chem. Commun.*, 2022, **58**, 4060–4062, DOI: https://doi.org/10.1039/D2CC00173J.

The authors regret that Alex L. Bagdasarian's name was spelled incorrectly in the original article. The correct author names are as presented here.

The Royal Society of Chemistry apologises for these errors and any consequent inconvenience to authors and readers.

## Supplementary Material

